# Pursuing policymakers, payors and public – expanding the beginning and end of the tuberculosis care cascade to reflect whole-of-society ambitions

**DOI:** 10.1371/journal.pgph.0006018

**Published:** 2026-03-18

**Authors:** Luan Nguyen Quang Vo, Ashna Ashesh, Ingrid Schoeman, Obioma Chijioke-Akaniro, Immaculate Kathure, Hoa Binh Nguyen, Anna K. Coussens, Hanif Esmail, Rein M. G. J. Houben

**Affiliations:** 1 Friends for International TB Relief, Ha Noi, Viet Nam; 2 Department of Global Public Health, Karolinska Institutet, Stockholm, Sweden; 3 Survivors Against TB, Delhi, India; 4 TB Proof, Cape Town, South Africa; 5 National Tuberculosis, Leprosy and Buruli Ulcer Control Programme, Federal Ministry of Health & Social Welfare, Abuja, Nigeria; 6 National Tuberculosis, Leprosy and Lung Disease Program, Ministry of Health, Nairobi, Kenya; 7 National Tuberculosis Control Programme, National Lung Hospital, Ha Noi, Viet Nam; 8 Wellcome Discovery Research Platforms in Infection, Centre for Infectious Diseases Research in Africa, Institute of Infectious Disease and Molecular Medicine and Department of Pathology, University of Cape Town, Cape Town, Republic of South Africa; 9 University College London, London, United Kingdom; 10 TB Modelling Group, TB Centre, London School of Hygiene and Tropical Medicine, London, United Kingdom; PLOS: Public Library of Science, UNITED STATES OF AMERICA

## Limitations of a unidimensional approach for a multifaceted disease

Significant progress has been made since tuberculosis (TB) was declared a public health emergency in 1993. Today’s ever-growing arsenal to screen, detect, and treat TB includes artificial intelligence-enhanced, handheld radiography, oral-swab-based, portable molecular diagnostics, and shorter treatments. These technological innovations are complemented by ambitious approaches such as community-wide screening (CWS), market-oriented private sector engagement and large-scale prevention campaigns. [1] Yet, gains towards ending TB remain insufficient and susceptible to shocks. [2]

A key obstacle to rapid progress is the historical focus on TB as a clinical issue, which has failed to effectively engage the broader audience beyond clinicians and researchers—particularly policymakers, payors and affected communities. A deadly consequence of not engaging policymakers and payors has been the lack of intentional policies that address the chronic lack of funding. This financing gap then impedes meaningful implementation of novel tools and approaches at scale. Meanwhile, not engaging affected communities results in lack of person-centered care, pervasive stigma, disconnected health system interventions, awareness gaps, and weak demand that impair health-seeking, contact tracing, treatment initiation and completion, and care for post-treatment TB sequelae. [3]

**Fig 1:**
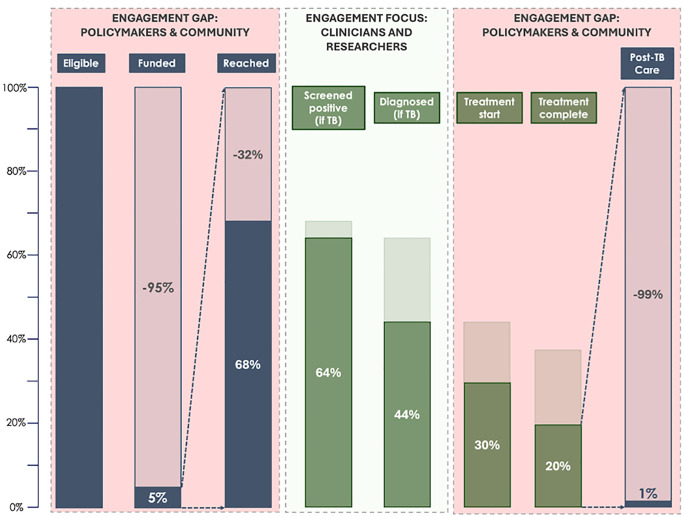
Policy cascade to reflect the whole-of-society approach to ending TB. Figure shows key steps in the expanded policy cascade. Green bars are classic clinical cascade, blue bars show extended policy cascade, including financing and community outreach (left) and post-TB care (right), For readability cascade is re-set at 100% after funding and before post-TB care. Shaded areas indicate where key interest-holders are fully engaged (‘Engagement focus’ - green) or where there is a gap (‘Engagement gap’ - pink). Values for the cascade sourced as follows: **Reached**: 68% in control group of the ACT3 trial who were reached for screening as reported by Marks et al. in the article “Community-wide Screening for Tuberculosis in a High-Prevalence Setting” published in the New England Journal of Medicine in 2019 (10.1056/NEJMoa1902129); **Screened positive**: 94% sensitivity on chest X-ray for any abnormality based on Table 3 on page 24 of the WHO consolidated guidelines on tuberculosis, Module 2: Screening, Systematic screening for tuberculosis disease. Geneva, Switzerland; 2021; **Diagnosed**: 69% diagnosed by WHO-recommended rapid diagnostic test - Xpert RIF/Ultra based on Table 3 on page 24 of the WHO consolidated guidelines on tuberculosis, Module 2: Screening, Systematic screening for tuberculosis disease. Geneva, Switzerland; 2021; **Treatment start**: 66% in the community recorded to initiate treatment as reported by Sifumba et al. in the article “Subclinical tuberculosis linkage to care and completion of treatment following community-based screening in rural South Africa” published in the journal BMC Global Public Health in 2024 (https://doi.org/10.1186/s44263-024-00059-0); **Treatment complete**: No evidence on treatment completion among those identified through systematic screening. If applied difference between reported and recorded treatment initiation from Sifumba et al (10.1186/s44263-024-00059-0), 66% would apply. Similarly, estimates from clinic-diagnosed individuals vary strongly. However, taking the mid-point between the lower bound of 50% in the private sector as reported by Lönnroth et al. in “Public-private mix for DOTS implementation: What makes it work?” published in the Bulletin of the World Health Organization in 2004 (S0042-96862004000800007) to 88% based on National TB Program reporting published in the WHO’s Global Tuberculosis Report 2025 would also fit with an estimated 66% overall; **Post-TB Care:** Set at 1% due to absence of validated or established treatments or guidelines. Dedicated post-TB care happening in isolated clinics (personal communication).

Frameworks such as the TB care cascade reflect the chronic exclusion of aforementioned key interest-holders. [4] This cascade traces the steps from persons screened to successfully completing treatment (Fig 1, green columns). Notably, this framework solely describes the clinical, programmatic pathway of disease, leaving substantial blind spots at the beginning and end of the care cascade (Fig 1, blue columns). These gaps epitomize the limited amount of financing available to reach the total population and minutia of people initiated on post-TB care. This exposes a strong selection bias in the population we target to provide care, so that even with unlimited funds and optimal use of the best tools, the impact on national burdens and the global epidemic would be negligible.

## A true point of beginning for TB

Traditionally, the first step in the cascade is the number of people screened for TB. However, this step reflects the obsolete approach of counting only those who seek care and whom we can afford to screen. These early gaps comprise ~95% of the population. [5] We need to face the uncomfortable reality that accepting anything less than the whole population as the true point of beginning for TB care contradicts our ambition to end TB. Closing these gaps would require strong engagement of non-traditional policymakers and payors such as finance, planning and investment ministries, members of parliament and appropriations committees or statutory and private insurance bodies as well as community leaders and civil society.

Recent developments in TB are illuminating a pathway to solve financing gaps. One trending topic entails the sustainable and innovative financing mechanisms for TB that have the potential to take the aforementioned innovations to scale. One particularly compelling approach entails the use of so-called sin taxes or solidarity subsidies to supplement TB budgets. [6] A successful example is the Philippines excise tax on tobacco and alcohol, which generates an annual ~USD5 billion for health, including TB. [7] Meanwhile, models estimated that ending TB via CWS in Vietnam would require investing only USD161 million for eight years. [8] This represents 1.15% of the country’s annual USD14 billion alcohol and tobacco market. Thus, a 10% or 20% sin tax could help end TB in 8 years, while also generating additional funds for reinvestment into post-TB care or other national priority areas such as elimination of hunger or eradication of poverty, which in conjunction would further reduce TB. [9]

The CAST/CAST-TB+ campaign for large-scale community-based screening in Uganda exemplifies the ambition to close the early gaps, reaching 6.4mn. Notably, a central tenet of the campaign was proactive outreach and engagement of community leaders, civil society, community health workers and volunteers. This enabled mass mobilization through stigma reduction, and transformed a government-led campaign into a collective mission, increasing trust in TB care providers, awareness of TB as a societal health challenge, and subsequent linkage to care. [10]

## All’s well that ends well

TB is a social disease, and progression, successful treatment and relapse-free recovery are strongly influenced by socio-environmental contributors. Thus, there has been a growing emphasis on person-centered care and addressing determinants such as undernutrition. [11] This emphasis has led to the development of the catastrophic cost indicator and the implementation of TB cost surveys. These have elucidated the harrowing reality that livelihood losses and paternalistic policies such as directly-observed therapy plunge half of all TB-affected families into poverty, who oftentimes are forced to liquidate productive assets or in extreme cases compelled to rely on crippling loans. [12] Consequently, persons unable to cope are forced to discontinue treatment.

Lesser known are TB’s long-term physical and psychological impairments on post-TB quality of life. [13] These can render survivors unable to pursue the same professions; incapable to complete the same recreational or routine activities; unwanted in the same romantic and familial relationships. These burdens have been long recognized, as the father of social medicine, Rudolf Virchow, 200 years ago posited “politics as medicine on a grand scale.” [14] Yet, they remain unseen by policymakers and payors, as our TB community has struggled to operationalize monitoring and evaluation frameworks for the economic and social impacts of TB that capture these measures within routine surveillance, resulting in persistently large post-TB treatment gaps.

Downstream indicators would incorporate the long-term disabilities and chronic nature of TB. One such step in the cascade could enumerate households experiencing catastrophic costs and benefitting from social support. Particularly regarding the latter, capturing these measures would require engagement of government agencies in labor, social health and welfare, or deployment of mechanisms grounded in citizen science and community-led monitoring. [15] A final downstream indicator is the proportion of TB survivors suffering from physical and psychological sequelae such as post-TB lung disease, and whether they receive care. For both steps, the current lack of engagement with TB communities for pre- and post-treatment support of TB patients is a glaring missed opportunity, as illustrated by the HIV community.

## Innovating the TB care cascade into a TB policy cascade

If the goal is to end TB, it is no longer sufficient to rely on a clinical care cascade that accounts only for people we can overtly afford to treat, and views treatment completion as the end of our event horizon. Cognizant that ending TB requires a whole-of-society approach, to return survivors to health we need a society-level cascade (Fig 1), which includes performance measures that hold to account our ability to involve policymakers and payors, and engage the community to close the massive gaps at the beginning and end of the cascade; to treat the whole population as the starting point and capture for how many people we had sufficient funding and outreach; and to account for the hidden costs of TB, whether this involves heavy economic losses or an impaired quality of life from TB-related sequelae post-treatment.

While the recent technology innovations have been exciting, it is vital we are innovative in our conceptualization of TB as a multidimensional clinical, political and social issue. This notion will require the engagement of key interest-holders up- and downstream of a policy cascade for TB, led by policymakers and payors, powered by affected communities. Only then will we access the necessary enablers—whether legislative, financial, social or cultural—to deploy ambitious strategies such as CWS at scale and have a shot at ending TB and its catastrophic impacts within our lifetimes.
